# Assessing eye health knowledge and practices amongst primary health care nurses in a rural district, South Africa  

**DOI:** 10.12688/f1000research.159132.1

**Published:** 2024-12-18

**Authors:** Lwandile Flatela, Zamadonda N Xulu-Kasaba

**Affiliations:** 1 Optometry, University of KwaZulu-Natal College of Health Sciences, Durban, KwaZulu-Natal, 4001, South Africa

**Keywords:** Visual impairment, Primary health care, Primary eye care, Nurses, Eye health, Primary health care clinics, knowledge and practices, rural eye health

## Abstract

**Background:**

The burden of visual impairment and avoidable blindness has been attributed to the shortage and poor distribution of adequately trained eye care personnel. In the district health system, primary health care (PHC) nurses are the first point of contact for eye care patients. To provide adequate eye care, the nurses in PHC clinics apply their knowledge on basic eye health to ensure best practices and management for patients that present at their clinics. Anecdotal evidence regarding the knowledge and practices of these PHC professionals is scarce. The purpose of this study was to evaluate PHC nurses’ knowledge and practices on eye health.

**Methods:**

A quantitative, cross-sectional study was conducted in a rural district of the Eastern Cape Province in South Africa. Stratified random sampling was used to select 28 from the 74 PHC clinics in the district. A researcher-administered questionnaire using a 5-point likert scale with options strongly disagree, disagree, neutral, agree and strongly agree, was developed, piloted and finalised for this study. The tool had four sections: demographic information, knowledge of eye health, practices on evaluating visual function and identification and management of presenting eye conditions. Data were collected, captured onto MSXcel, cleaned, coded and analysed descriptively, and for Chi-squared significance at 95%, using R statistical software.

**Results:**

From 200 responses, 86.4% (n=172) were females of the African race. Most (93.5%, n=187) had never undergone formal training in eye care. Only 28% (n=56) correctly identified a mature cataract while 28% (n=56) correctly identified signs of glaucoma; the global leading causes of preventable blindness. A total of 94% (n=188) failed to accurately measure visual acuity on a patient.

**Conclusions:**

PHC nurses showed poor knowledge and practices of common eye conditions. There is an urgent need for eye health training for PHC nurses, and the clear management protocols on eye care.

## Introduction

Visual Impairment (VI) remains a major public health concern that continues to rise. ^1^ Globally, an estimated 596 million people have reduced vision at distance, while more than 500 million suffer from uncorrected near vision. ^2^ According to the World Health Organisation (WHO), 80% of all VI is preventable, while 90% is found in low-to-middle income countries (LMIC). ^3^ It is further estimated that 76% of moderate and severe VI cases in southern Sub-Saharan Africa (SSA) are caused by cataract, glaucoma and uncorrected refractive error (URE). ^3, 4^ Of further concern are the dire socio-economic consequences of VI suffered by the affected individuals. ^5^

Primary eye care (PEC) is one of the pillars of primary health care (PHC) recommended by the WHO. ^6^ PEC involves eye health promotion, treatment of simple eye diseases, and identification and prompt treatment of persons needing eye care ^6^; all areas that are pivotal in preventing blindness and VI. Poor and rural populations bear the burden of VI and its consequences as resources are skewed against these communities. ^4^ Amongst other challenges, poor access to clinics with eye health services, weak infrastructure, and a shortage of human resources amplify the burden of VI in LMIC. ^7– 10^ Blanchet *et al* emphasise that poor integration of eye health services into the health systems and scarcity of eye health policies in LMICs have a negative contribution towards achieving improvement in visual health. ^11^ This would further impede the attainment of the third Sustainable Development Goal centred on good health and vision. ^12^

In South Africa’s district health system (DHS), the majority of patients needing PEC initially present themselves at PHC centres. ^13^ The level of care provided has been described as inadequate in relation to community needs, and inferior to the standard prescribed by the National Department of Health (NDoH). ^14, 15^ As PHC workers are the first point of contact with patients, they are tasked with providing PEC to the communities in which they are based. To adequately deliver PEC services, PHC providers are tasked with the responsibility of identifying, managing and referring patients accordingly and timeously. ^7^ There is a dearth of evidence evaluating their knowledge and practices in basic eye care services in the rural areas of SSA countries such as South Africa. ^16^ This study aimed to assess PHC nurses’ knowledge and practices in PEC in order to evaluate how eye patients are being managed in PHC clinics within the district and other similar rural contexts.

## Methods

### Study setting

Alfred Nzo District Municipality (AND) is the smallest of eight districts in the Eastern Cape province of South Africa. With 867 864 inhabitants (12.2% of the province’s population), ^17^ AND is one of the most densely populated districts in the province. With a density of 81 people/ km ^2^. ^17^ AND is also the most impoverished, rural, and underdeveloped district in the province, coupled with a high unemployment rate. ^18^ The district has four local municipalities, with 74 PHC clinics. To contrast, this is a higher density than Umkhanyakude District, in the neighbouring KwaZulu-Natal province, which is also rural with 625 846 inhabitants, and a density of 49 people/km ^2^. ^19^

### Study design

A cross-sectional study was conducted in 28 randomly selected Primary Health care clinics (PHCC) within the AND. A sample size was calculated using the single population proportion formula, ^20^ where significance was at the 95% confidence level and 5% degree of precision. A figure of 50% was used for the expected knowledge base. District office registers, reporting on staff numbers and clinic contact details, were obtained from the AND office to ascertain a population size. These were approximately ten years old and confirmed a nursing workforce of 935 nurses in the PHCC within the district. There was no available updated version that accounted for deaths, retirements, and resignations. Factors such as retirement, deceased employees, and transfers away from the district, promotions, unfilled vacant posts, and the like were reasons given by clinic supervisors for actual staffing numbers being approximately 50% of that which had been anticipated according to the registers. To establish a more accurate sample size, the Principal Investigator (PI) telephonically communicated with the heads of each clinic, making appointments in advance to visit them for stakeholder engagement in preparation for data collection. On arrival at the clinics, it was apparent that the actual staffing was approximately 50% of that which had been anticipated according to the registers. As such, the population (N) was adjusted to 50% of 935 where N = 466.

Using the single population proportion formula, the minimum sample size calculation used was:

$$n=\frac{\frac{Z^{2}p\left(1-p\right)}{d^{2}}}{1+\frac{Z^{2}p\left(1-p\right)}{{\mathrm{Nd}}^{2}}}$$

z (for a confidence level of 95%, standard normal distribution) = 1.96

d (acceptable/tolerated margin of error of 5%) = 0.05

p (if no prevalence/prior study the proportion is set at 50%) = 0.50

N (population size) = 466

The recommended sample size was calculated, and a 5% error margin was further added to the sample size to produce a required minimum sample size (n) of 211.

Stratified random sampling was applied across the district to ensure even representation of the various municipalities. Proportioning of participants was applied to match the distribution of clinics in the various municipalities. Participant numbers were 35% (n = 70) from Mbizana, 15% (n = 30) from Ntabankulu, 35% (n = 70) from Mzimvubu and the remaining 15% (n = 30) from Matatiele (15%).

### Data collection, processing and analysis

The Principal Investigator (PI) contacted the heads of each clinic again, making appointments in advance to visit them for data collection purposes. Low staff numbers were found at most sites due absenteeism (due to quarantine or contact tracing guidelines resulting from the COVID-19 pandemic), leave and illness. From the available workforce, the PI addressed the staff in their common room to inform them of study aims and voluntary participation, only the consenting PHC staff were enrolled in the study. Furthermore, some PHC staff members who had been enrolled, ultimately did not participate, citing the high numbers of patients that were still waiting to be seen during business hours. The final number of participants who were enrolled in the study was 200. Willingness of voluntary participation was confirmed by signing of the consent forms by each willing participant.

The contents of the data collection tool were informed by various primary eye health service documents and screening guidelines for ocular health in South Africa, Nigeria, Uganda and a few other African countries. ^5^ Data collection tools from previous studies that had assessed primary eye care skills amongst nurses and community health workers within sub-Saharan Africa (SSA) were also used to compile the final questionnaire that was used in this study. ^21^ After completion, the comprehensive questionnaire was piloted on 20 PHC nurses in another province, where input for improvement was requested and given. None of the original questions were removed, instead the wording was simplified in some for ease of understanding. After modification, the PI finalised the tool for use in the study. The tool was divided into three sections: Part 1 - demographic data; Parts 2 & 3 - knowledge of different eye health professions and identification of common eye conditions using coloured pictures and brief descriptions of defining symptoms, and Part 4 focused on practices through task observation of the participants by the PI who sat as a patient for all the participants when they performed the task. Participants were provided with a distance and near tumbling E chart and a tape measure for distance measurement. Here the PI scored each participant on their ability to perform a visual acuity measurement and thereafter record the findings. A marking guide was derived and used for this evaluation ( Table 1), and participants were considered to be competent if they obtained a minimum score of seven out of ten (70%).

**Table 1.** Scoring guide for VA assessment.

Task	Points awarded for correct practice	Points awarded for incorrect answer
Distance from VA chart	2	0
Accurate instructions to patient	1	0
Ensuring sufficient lighting	1	0
Monocular execution of assessment	2	0
Seating patient and chart at the same height	1	0
Correct near VA target distance	1	0
Accurate recording of distance and near VA	2	0

Due to poor internet connectivity issues, hard copies of the researcher-administered data collection tool were used. Each willing participant was individually invited into the tea room by the PI. The PI sat with one participant at a time, and guided on completion of the questionnaire. Where translation was requested, the PI assisted as he is conversant in the local language. After completion of the questionnaire, the PI asked the participants to measure distance and near visual acuity on him away from other staff members. Data was collected manually from all participants, captured onto Microsoft Excel, cleaned, coded, stored, and transferred to the R software for storage. Data were analysed descriptively for frequencies and chi-squared significance at the 95% confidence index.

## Results

### Demographic and training information

A 95% response rate (n = 200) was achieved, and participants were from 28 of the 74 PHC facilities across AND. The majority of 172 (86.4%) were female. All participants were from the African race, where 96% spoke the IsiXhosa language, and the remaining 4% spoke IsiZulu. Of further concern, is the fact that the majority (93.5 %) have never been formally trained in eye care. Further demographic details have been detailed in Table 2.

**Table 2.** Demographic information and professional training characteristics.

Characteristic	Frequency (n)	Percentage (%)
**Age (in years)**		
1. <30	27	13.5
2. 30-39	48	24
**3. 40-49**	**68**	**34**
4. 50-59	24	12
5. >60	33	16.5
**Job Title/Category**		
**1. Professional nurse**	**154**	**77**
2. Enrolled nurse	15	7.5
3. Enrolled nursing assistant	29	14.5
4. Ophthalmic nurse	2	1.0
**Highest qualification**		
1. Basic 1 or 2 years nursing training	43	21.5
**2. Nursing Diploma/Nursing Degree/Post basic qualification**	**157**	**78.5**
**Duration of Service (in years)**		
**1. 0 – 5**	**79**	**40**
2. 6 – 15	78	39
3. 16 – 25	26	13
4. >25	17	8
**Trained in Eye care**		
1. No	**187**	**93.5**
2. Yes	13	6.5
**Institutions where staff were trained in eye health**		
1. Public Tertiary institution	4	2
2. NGO	4	2
3. DoH	5	2.5
4. I was never trained on eye health	**187**	**93.5**
**Completed refresher eye care training**		
**1. No**	**195**	**97.5**
2. Yes	5	2.5
**Refresher eye care training frequency**		
1. Every two years	1	0.5
2. Annually	2	1
3. Other	2	1
**4. Never**	**195**	**97.5**

None of the visited PHCC had a Snellen or other visual acuity (VA) chart or a penlight torch. The PI took his own equipment to the sites for assessment purposes. Additionally, none of the surveyed facilities across the district had a staff member responsible for ophthalmic care, and none of the facilities had a designated eye care consulting room. Only 35.5% of the participants affirmed that they understand the management and treatment protocols for eye health.

Knowledge of PHC nurses on different eye professionals ( Table 3).

**Table 3.** Basic knowledge on roles of different Human resources for eye health (HReH).

Knowledge analysis		
	Disagree	Agree
I know the different types of eye health professionals (p = 0.322)	46%	54%
Optometrists are mainly trained in cutting lenses ( **p < 0.001**)	35%	65%
Ophthalmic Nurses train for a further 2 years (after basic nursing training) to qualify and practice in that role ( **p = 0.024**)	42%	58%
Ophthalmic Nurses are best suited to work in PHC settings ( **p < 0.001**)	28%	72%
Optometrists are best suited to only work in tertiary settings ( **p < 0.001**)	32%	68%
Ophthalmologists are best trained in refraction and low vision services ( **p = 0.024**)	42%	58%

Practices in the measurement of VA ( Table 4).

**Table 4.** Showing participants’ scores on VA measurement.

Practice analysis			
Total score achieved	% of scores	Nr of participants who achieved scores	Final outcome summarised
**10**	**0.0%**	**0**	6% passed
**9**	**1.0%**	**2**	
**8**	**1.5%**	**3**	
**7**	**3.5%**	**7**	
6	**11.5%**	23	94% failed
5	**22.5%**	45	
4	**30.5%**	61	
3	**15.5%**	31	
2	**14.0%**	28	
1	**0.0%**	0	
0	**0.0%**	0	

Knowledge and practices of different eye conditions and ocular pathologies ( Table 5).

**Table 5.** Showing levels of identification of conditions (from coloured pictures) and management thereof. Knowledge practices and case management with common visual conditions.

Knowledge and practical analysis				
Agreement responses by participants are recorded below	KNOWLEDGE (CORRECTLY IDENTIFIED)		PRACTICE (CORRECT MANAGEMENT)	
	%	P-VALUE	%	P-VALUE
**CATARACT**				
**K**: Accurate identification of mature cataract from coloured picture.	28	**<0.001**		
**P**: A patient with (a mature cataract) must be transferred to hospital urgently.			26	**<0.001**
**UVEITIS**				
**K**: Accurate identification of uveitis from coloured picture and symptom description.	16	**<0.001**		
**P**: A patient with (uveitis) needs an urgent referral.			32	**<0.001**
**VERNAL KERATOCONJUNCTIVITIS (VKC)**				
**K**: Accurate identification of VKC picture and case history.	20	**<0.001**		
**P**: A patient with VKC cannot be managed in the clinic and should be referred urgently to a hospital.			24	**<0.001**
**PRIMARY OPEN ANGLE GLAUCOMA**				
**K**: Accurate identification of glaucoma from description of throbbing eye pain, family history and visual field loss.	28	**<0.001**		
**P**: This patient (with glaucoma) does not need any referral; they need to be monitored yearly.			40	0.016
**PRESBYOPIA**				
**K**: Accurate identification from symptoms and case history.	60	0.007		
**P**: Accurate management based on case history and symptoms.			42	**<0.001**
**CORNEAL FOREIGN BODY**				
**K**: Accurate identification based on coloured picture and case history.	25	0.007		
**P**: Good management and urgent referral based on case history and symptoms.			25	**<0.001**

Key: **K** - Knowledge, **P** – Practices.

## Discussion

This study sought to evaluate PEC knowledge amongst the PHC nurses in AND, and further assess the current practices and protocols that are used in managing eye care cases. As most patients initially present at PHCC for their health concerns, including eye health, it is critical to assess the knowledge and practices of nurses that manage the larger population at those health facilities.

In our study the most common cadre of staff that participated were professional nurses of the female gender aged 41 to 50 years of age. This finding is similar to studies in Nigeria and other parts of South Africa, where most participants were females in the same age grouping. ^7, 22^ The female bias is to be expected as the nursing profession was a predominantly female career option, and viewed as an extension of the domestic role played by women in homes and other sectors of society. ^23, 24^ Additionally, its common traits of caregiving and nurturing also align nursing more with female social gender roles than male gender roles. Similarly, the age range of most participants is within the most common labour force in South Africa, as evidenced by Statistics South Africa in their reports. ^25^ An overwhelming majority of the participants had a service record of 15 years, meaning that they had a fairly good idea of the PHC environment, with another two decades of service prior to their retirement. As such, they were within their earlier years of service and were still amenable to learning and skill development for the betterment of their careers.

It is satisfactory to learn that the majority of the participants were trained and qualified as professional nurses. The AND fares well in this regard as the South African Nursing Council (SANC) distribution report shows that the Eastern Cape province has an average of 61 % of the nursing staff trained as professional nurses, ^26^ one of the higher prevalence’s in the country. This could be attributed to the many nurse training facilities that exist in the Eastern Cape Province and is a positive case for general public health in the communities they serve. The fact that all the participants spoke isiXhosa, the local language in the province was an indicator of a homogenous culture of the participants. In light of this, it will benefit learning if refresher courses use training material in this language. ^27^ Previous evidence has affirmed that medical learning is positively enhanced when a mother tongue or native language is used. ^26^

Of significant concern, however, is the markéd shortage of Human Resources for Eye Health (HReH) in the PHCC, which was confirmed by the far ‘lower-than-expected’ numbers of study participants on arrival at the clinics. Staffing numbers at PHCCs were less than half of the numbers confirmed by the regulating office, and some clinics had a single nursing professional on duty for the day, tasked with seeing to the general and eye care of the entire community. Health professional shortages are a serious concern in Africa, worsened by the “brain drain”, or exodus of experienced HReH from poorer countries to economically affluent first world countries. ^28^ This further exacerbates prevailing health system challenges and worsens the burden of disease in poorly resourced and underdeveloped African countries. Professional nurses are amongst the highest numbers of trained health professionals that are known to have left South Africa in search of improved working conditions, better staffing and financial rewards offered by first world economies. ^29^ In communities such as the AND, this shortage is a reality that further marginalises the population, and impedes the delivery of basic eye care in their clinics.

The fact that nearly none of the employees at the PHCCs had undergone eye health training further intensifies the severe staffing deficit. The number of participants was lessened by the fact that the original sample size was incorrect, owing to poor record keeping. In many health sectors, it has been argued that good record keeping is associated with appropriate standards of nursing, improved patient care, and swift reaction in the instance of emerging legal issues. ^30– 32^ It is imperative that this be improved for clear, transparent data and reporting in the future. In this study, this was seen as a significant part of the dire issues that add to the barriers to the delivery of adequate eye care to communities in the AND. Policymakers who use the staffing numbers that the district office has are under the impression that the district’s PHCC are well staffed, which is contrary to the reality of the situation on the ground. Consequently, this insufficient staffing worsens the burden of diseases, increases VI and the prevalence of avoidable blindness, and negates the achievement of universal health coverage through reduced service delivery, realised due to staffing shortages. The district needs to improve its staffing of eye clinics and its record keeping to ensure accessibility of sufficient eye care. Better access to eye care will further ensure improved learning opportunities for children through the school health system, improved employability opportunities for young adults, and subsequently reduced levels of poverty in this region.

### Eye health knowledge

PHC workers are the first point of care for many rural South African communities and the first point of contact for health conditions in developing countries. ^21^ There is an expected level of knowledge and skill that is required in order to provide effective management of eye conditions, and ensure that preventable blindness is addressed timeously. ^33^

Table 3 shows that almost half (46%) of the respondents did not know the basic roles and competencies of the different HReH, which indirectly affects their referral practices, as good knowledge of these would enable them to adequately triage ocular cases to the correct HReH. In knowing what the key roles of ophthalmologists, optometrists, opticians and ophthalmic nurses are, the referral of cases that could not be managed in the PHCC would be accurate with a reduced waiting period for the patient.

Knowledge of common eye conditions were also very weak. Similar results were found in Pakistan and Ethiopia, where poor knowledge of basic ocular conditions was noted amongst various health professionals. ^15^ Reasons expressed for this poor knowledge was the fact that in their training, general practitioners spend a maximum of four weeks only in the ophthalmology rotation, which is a very short training period for one to gain sufficient knowledge and information on this area of healthcare. ^34, 35^ Similarly, with nurse training in South Africa, there is no clinical component in ophthalmology or optometry, which is probably why the knowledge levels in this study were very poor. Previous studies have also shown that inadequate eye care knowledge, demonstrated by nurses, reduced their confidence in dealing with patients with eye problems. ^35, 36^ Additionally, the clinical knowledge of basic ocular conditions, and leading causes of blindness and visual impairment were poorly identified and managed by the respondents in the AND.

Cataract is the leading cause of preventable blindness in SSA and the world, and an easily identifiable condition; however, approximately one quarter of the respondents identified it correctly. Worse still, the management of this condition was seen as urgent and immediate, which is inaccurate, and the cause of unnecessarily overloading referral transport and overloading an already strained system that could have given the ambulance space to a more deserving patient. This prevalence is significantly lower than that found in a similar study in Nepal where most PHC workers were able to identify a cataract accurately. ^37^ Similarly in other studies within African countries: Tanzania, Malawi, Kenya and Nigeria, PHC nurses were more knowledgeable in identifying and managing patients with cataract, VKC and glaucoma. ^38, 39^ Foreign body sensations were the only condition that although not accurately identified, was managed adequately in this study. This was probably due to the discomfort, pain, and urgency that patients present with. In KwaZulu Natal Province, South Africa, knowledge regarding primary eye care was found to be relatively good amongst participants in PHCs within hospitals. ^15^

This was probably due to the fact that hospitals employ ophthalmic nurses and optometrists in their primary care and outpatient departments. Unlike optometrists and ophthalmic nurses, most of the PHC nurses in this study were not HReH trained in eye health.

Poor knowledge and management of patients by PHC nurses are concerning factors as they marginalise patients in poor, rural communities, deter universal eye health access, and exacerbate the burden of disease. Clinic PHC nurses should be well-versed in eye care to protect patients, avoid ocular complications and adequately address ocular problems as this is all directly linked to visual impairment. ^40^ Health service managers should look to improving this as it has far-reaching effects on community wellbeing and general health.

### Training in eye care

Most of the participants in our study reported to have never had any eye health training. The very few who have received proper training had not had a refresher course. As a result, the majority of eye care cases were then referred, even those that could have been managed timeously and locally. There were poor management guidelines in place and no set referral criteria used. In agreement, previous studies reported inappropriate referral pathways in northern Nigeria ^39^ while, to the contrary, evidence reported a clear and precise referral pathway in India. ^41^

This low number of trained PHC workers in PEC is in line with a study in Nigeria that found that not even one of the respondents had received training on PEC in their facility. ^42^ On the contrary, in a similar study done in Kenya it was found that a much higher proportion (two thirds) of the participants in PHCC had received PEC training. ^38^ It stood to reason that the Kenyan study reported that almost all PHC workers provided adequate eye care with confidence, which could be cited as an example of competence overreaching as those that were not trained learned from the others and managed patients satisfactorily. ^38, 43^ In this study, the extremely low number of PHC nurses who had received some form of eye health training could be linked to the basic nursing curriculum – which does not mention eye health – and lack of continued professional development in the nursing profession. The majority of those who had received training in eye care had never completed any refresher eye care training. In our study, only two of the respondents were ophthalmic nurses, and both were working as professional nurses due to a lack of specialised post funding at clinic level. To further exacerbate the problem is the fact that only three nursing colleges offer ophthalmic nurse specialisation training in the entire country. Some non-governmental organisations (NGO’s) like The Fred Hollows Foundation have tried to bridge this gap by offering some kind of eye care training for PHC workers; however, this has not been sustained leading to its eventual failure.

It is clear that nurse training needs to better embrace eye health training so as to effectively manage cases in the PHCC. Poor identification of leading causes of blindness and visual impairment, poor knowledge of HReH and poor management of eye health cases all point to much needed training and refresher training of nurses in eye health. This needs to be addressed with a view to providing eye health training.

### Clinical skill observation and practice norms

The study results show that the ability to carry out a visual acuity assessment by the PHC workers in the AND is far below the expected level ( Table 4). A great majority of the participants did not meet the minimum competency levels of this clinical technique and scored less than 7/10 on assessment. Visual acuity is an important measure of visual function and is necessary for decision making with ophthalmic patients. Visual acuity is the most frequently performed measure of visual function in clinical practice and most people worldwide living with VI are living in low and middle-income countries. ^44^ Visual acuity measurements then ought to be routinely conducted with all patients visiting the PHC clinic. ^7^ Inaccurate execution, as displayed by those in this study, further marginalises scholars and older patients who could have played a significant role in society had the visual impairment been assessed accurately, and visual assistive devices or treatment initiated for improved vision. Similarly in studies in Nepal and Eastern Africa, only 14% and 12.3% of the PHC workforce respectively could measure visual acuity. ^7, 37^ Furthermore, the poor outcome in our study is also not surprising considering that none of the visited PHC clinics had a Snellen chart to measure patients’ visual acuity.

### Limitations

The study location was in a deep rural area with an extremely poor roads network, making it difficult to reach the clinics on the extreme periphery of the region. Inclusion of these facilities may have added substantial information to the study. A general shortage in staffing was, however, noted in the clinics where staffing ranged from one PHC worker on duty, where the study could not be prioritised over patients that were waiting to be served. In PHCCs such as this, we were forced to help where we could and turn back as participation in the study was not possible. It would be beneficial for the study to be conducted in more districts to get a better understanding of the knowledge and practices of PHC nurses on a larger scale.

### Implications of study

Data collected and analysed in the study sets a clear basis on the need for training and development of eye health knowledge in PHCC such as this one, more especially the ones in rural settings where local services are a dire need, due to financial limitations. It is imperative that training insitutions prioritise developing materials to meet this need and empower HReH in PHCC.

### Recommendation

We recommend basic eye health training to be included in the nursing curriculum to equip PHC workers with the necessary skills to improve their role in VI. Refresher courses are recommended for staff in PHCC to ensure adequate skill levels in their clinics. Training materials should be provided in the local language to enhance the homogenous culture in this province. Secondly, there needs to be an establishment of guidelines and funnelling protocols for eye health cases. HReH posts must be created, funded, and filled for sufficient staffing and effective eye care.

## Conclusion

Most of the nurses in this district are females within a suitable working age, who speak the local language spoken by patients in that district. This study showed that the PHC nurses in the Alfred Nzo region had inadequate eye health knowledge on HReH and basic eye conditions that are leading causes of visual impairment and blindness globally. This has a grave impact on the treatment, referral and management of treatable and preventable visual impairment conditions in the region. PHC nurses in this area did not know how to measure visual acuity. Most nurses were never trained in eye health. None of the clinics visited had a Snellen chart for visual acuity measurement. There were no basic guidelines and referral protocols that could be referred to for eye conditions. Eye health is clearly not prioritised in this district. Health managers need to prioritise eye health, specifically PEC, and seek to train nurses in order to provide adequate universal eye health within the communities they work.

## Declarations

### Ethics approval

Written ethical clearance was obtained from UKZN BREC/00000663/2019 and gatekeeper permission was obtained from the DoH, Alfred Nzo District, and the DoH, Eastern Cape Province.

## Consent to participate

Informed written consent was obtained from all participants included in the study.

## Author contributions

Detailed contributions from the authors have been tabled in Table 6 below.

**Table 6.** Authors’ contributions.

Area of contribution	Contributor
Conceptualisation	ZXK
Data curation	LBF & ZXK
Formal analysis	LBF & ZXK
Funding acquisition	No funding was received.
Investigation	LBF
Methodology	LBF & ZXK
Project administration	LBF
Resources	LBF & ZXK
Software	LBF & ZXK
Supervision	ZXK
Validation	LBF & ZXK
Visualization	LBF & ZXK
Writing – original draft preparation	LBF & ZXK
Writing – review & editing	ZXK
LBF – Lwandile B Flatela	
ZXK – Zamadonda Xulu-Kasaba	

## Ethical considerations

Following ethical clearance by the Biomedical Research Ethics Committee (BREC/00000663/2019) at the University of KwaZulu-Natal in May 2020, and gatekeeper permission by the Eastern Cape Department of Health (DoH), data collection was delayed by the national lockdown imposed by the COVID-19 pandemic. When restrictions were lifted, re-certification was obtained, and data collected from February to June 2021.

## Data Availability

Data in the form of questionnaire response spreadsheets and ethical permissions is available on Zenodo underproject name “Assessing eye health knowledge and practices amongst primary health care nurses in a rural district, South Africa” at https://doi.org/10.5281/zenodo.14150666. All extended data is available under the terms of the Creative Commons Zero “No rights reserved” data waiver (CC0 1.0 Public domain dedication). Access to this dataset requires registration with an IEEE account, which is free.

## References

[1] Holden BA Fricke TR Wilson DA : Global prevalence of myopia and high myopia and temporal trends from 2000 through 2050. *Ophthalmology.* 2016;123(5):1036–1042. 10.1016/j.ophtha.2016.01.006 26875007

[2] Burton MJ Ramke J Marques AP : The Lancet global health Commission on global eye health: vision beyond 2020. *Lancet Glob. Health.* 2021;9(4):e489–e551. 10.1016/S2214-109X(20)30488-5 33607016 PMC7966694

[3] Flaxman SR Bourne RR Resnikoff S : Global causes of blindness and distance vision impairment 1990–2020: a systematic review and meta-analysis. *Lancet Glob. Health.* 2017;5(12):e1221–e1234. 10.1016/S2214-109X(17)30393-5 29032195

[4] Lilian RR Railton J Schaftenaar E : Strengthening primary eye care in South Africa: an assessment of services and prospective evaluation of a health systems support package. *PLoS One.* 2018;13(5):e0197432. 10.1371/journal.pone.0197432 29758069 PMC5951550

[5] Marques AP Ramke J Cairns J : The economics of vision impairment and its leading causes: A systematic review. *EClinicalMedicine.* 2022;46:101354. 10.1016/j.eclinm.2022.101354 35340626 PMC8943414

[6] Ekpenyong BN Osuchukwu N Ndep O : Ophthalmic Skills Assessment of Primary Health Care Workers at Primary Health Care Facilities in Rural Communities in Cross River State, Nigeria. *J. Niger. Optom. Assoc.* 2018;20(1):55–59.

[7] Kuper H Polack S Eusebio C : A case-control study to assess the relationship between poverty and visual impairment from cataract in Kenya, the Philippines, and Bangladesh. *PLoS Med.* 2008;5(12):e244. 10.1371/journal.pmed.0050244 19090614 PMC2602716

[8] Bechange S Jolley E Virendrakumar B : Strengths and weaknesses of eye care services in sub-Saharan Africa: a meta-synthesis of eye health system assessments. *BMC Health Serv. Res.* 2020;20(1):1–8. 10.1186/s12913-020-05279-2 PMC7203845 32375761

[9] World Health Organization: Universal eye health: a global action plan 2014-2019. 2013.

[10] Gooding K : Poverty and blindness: a survey of the literature. *Sightsavers International.* 2006.

[11] Blanchet K Gilbert C Savigny D de : Rethinking eye health systems to achieve universal coverage: the role of research. *Br. J. Ophthalmol.* 2014;98(10):1325–1328. 10.1136/bjophthalmol-2013-303905 24990874 PMC4174128

[12] The United Nations Development Programme: Sustainable Development Goals. 2015. [Accessed 12 November 2023]. Reference Source

[13] Fusheini A Eyles J : Achieving universal health coverage in South Africa through a district health system approach: conflicting ideologies of health care provision. *BMC Health Serv. Res.* 2016;16(1):1–11. 10.1186/s12913-016-1797-4 27717353 PMC5054620

[14] Naidoo K : Poverty and blindness in Africa. *Clin. Exp. Optom.* 2007;90(6):415–421. 10.1111/j.1444-0938.2007.00197.x 17958563

[15] Xulu-Kasaba Z Mashige K Naidoo K : Knowledge, Attitudes and Practices of Eye Health among Public Sector Eye Health Workers in South Africa. *Int. J. Environ. Res. Public Health.* 2021;18(23):12513. 10.3390/ijerph182312513 34886238 PMC8656467

[16] Courtright P Mathenge W Kello AB : Setting targets for human resources for eye health in sub-Saharan Africa: what evidence should be used? *Hum. Resour. Health.* 2016;14(1):1–8. 26984773 10.1186/s12960-016-0107-x PMC4794905

[17] Statistics South Africa: Statistics South Africa Eastern Cape.pdf. 2018.

[18] Alfred Nzo district municipality 2024. Reference Source

[19] Umkhanyakude District Municipality 2024. Reference Source

[20] Cochran WG : *Sampling techniques.* John Wiley & Sons;2007.

[21] Byamukama E Courtright P : Knowledge, skills, and productivity in primary eye care among health workers in Tanzania: need for reassessment of expectations? *Int. Health.* 2010;2(4):247–252. 10.1016/j.inhe.2010.07.008 24037865

[22] Joubert P : Nurse Shortage in South Africa Nurse/Patient Ratios. *Report by Solidarity Research Department, Addendum.* 2009;5(4):3.

[23] Trotter LJ : Making a career: Reproducing gender within a predominately female profession. *Gend. Soc.* 2017;31(4):503–525. 10.1177/0891243217716115

[24] Evans J : Men nurses: a historical and feminist perspective. *J. Adv. Nurs.* 2004;47(3):321–328. 10.1111/j.1365-2648.2004.03096.x 15238127

[25] Stats SA: CENSUS 2022 2023 [30/11/2023]. Reference Source

[26] South African Nursing Council: *Provincial distribution of nursing manpower versus the population of South Africa.* Pretoria:2022.

[27] Alsuliman T Alasadi L Mouki A : Language of written medical educational materials for non-English speaking populations: an evaluation of a simplified bi-lingual approach. *BMC Med. Educ.* 2019;19(1):418. 10.1186/s12909-019-1846-x 31711476 PMC6849289

[28] Zimbudzi E : Stemming the impact of health professional brain drain from Africa: a systemic review of policy options. *J. Public Health Afr.* 2013;4(1):4. 10.4081/jphia.2013.e4 28299093 PMC5345423

[29] Aikman N : The crisis within the South African healthcare system: A multifactorial disorder. *S. Afr. J. Bioeth. Law.* 2019;12(2):52–56. 10.7196/SAJBL.2019.v12i2.694

[30] Griffiths P Debbage S Smith A : A comprehensive audit of nursing record keeping practice. *Br. J. Nurs.* 2007;16:1324–1327. 10.12968/bjon.2007.16.21.27718 18073669

[31] McGeehan R : Best practice in record-keeping. *Nursing Standard (through 2013).* 2007;21:51–55. 10.7748/ns.21.17.51.s54 17260938

[32] Wood C : The importance of good record-keeping for nurses. *Nurs. Times.* 2003;99:26–27. 12599905

[33] Aghaji AE Gilbert C Ihebuzor N : Strengths, challenges and opportunities of implementing primary eye care in Nigeria. *BMJ Glob. Health.* 2018;3(6):e000846. 10.1136/bmjgh-2018-000846 30613423 PMC6304095

[34] Fatima I Ahmad I : Knowledge, Attitude, Practice (KAP) study regarding optometric services among general practitioners in Lahore. *Ophthalmol. Pak.* 2018;8(01):14–17.

[35] Jaafar S Al-Jubouri MB Alfatlawee DM : Nurses’ Knowledge based on Evidence Based Practice toward Eye Care for Intensive Care Units Patients. *Indian J. Forensic Med. Toxicol.* 2020;14:1314–1318.

[36] Khalil N Abd Elhameed S Abdel-Kader F : Critical care nurses’ knowledge and practices concerning eye care of patients at two teaching university hospitals, Egypt. *Nurs. Healthcare Int. J.* 2019;3:000188. 10.23880/NHIJ-16000188

[37] Burn H Puri L Roshan A : Primary eye care in eastern Nepal. *Ophthalmic Epidemiol.* 2020;27(3):165–176. 10.1080/09286586.2019.1702217 31842661 PMC7114913

[38] Kalua K Gichangi M Barassa E : Skills of general health workers in primary eye care in Kenya, Malawi and Tanzania. *Hum. Resour. Health.* 2014;12(1):1–6. 25860909 10.1186/1478-4491-12-S1-S2 PMC4108885

[39] AbdulRahman AA Rabiu MM Alhassan MB : Knowledge and practice of primary eye care among primary healthcare workers in northern Nigeria. *Trop. Med. Int. Health.* 2015;20(6):766–772. 10.1111/tmi.12486 25708905

[40] Naidoo K : Poverty and blindness in Africa. *Clin. Exp. Optom.* 2007;90(6):415–421. 10.1111/j.1444-0938.2007.00197.x 17958563

[41] Rao GN Khanna RC Athota SM : Integrated model of primary and secondary eye care for underserved rural areas: the LV Prasad Eye Institute experience. *Indian J. Ophthalmol.* 2012;60(5):396–400. 10.4103/0301-4738.100533 22944748 PMC3491264

[42] Onakpoya O Adeoye A Adegbehingbe B : Assessment of human and material resources available for primary eye-care delivery in rural communities of southwestern Nigeria. *West Indian Med. J.* 2009;58(5):472–475. 20441068

[43] Walter ND Lyimo T Skarbinski J : Why first-level health workers fail to follow guidelines for managing severe disease in children in the Coast Region, the United Republic of Tanzania. *Bull. World Health Organ.* 2009;87(2):99–108. 10.2471/BLT.08.050740 19274361 PMC2636200

[44] Bastawrous A Rono HK Livingstone IA : Development and validation of a smartphone- based visual acuity test (peek acuity) for clinical practice and community-based fieldwork. *JAMA Ophthalmol.* 2015;133(8):930–937. 10.1001/jamaophthalmol.2015.1468 26022921 PMC5321502

