# Supplementary material for: Assessing eye health knowledge and practices amongst primary health care nurses in a rural district, South Africa  
Source: F1000Res. 2026 Jan 24;13:1530. Originally published 2024 Dec 18. [Version 2] doi: 10.12688/f1000research.159132.2 (PMC12853019; doi:10.12688/f1000research.159132.2)
Supplement: Peer review history

# Reviewer report: Reviewer response for version 2

**DOI:** 10.5256/f1000research.195419.r452909

**Published:** 2026-01-28

**Authors:** Michael Bowen

**Affiliations:** 1 The College of Optometrists, London, England, UK

These are helpful amendments and the paper is now much clearer and more accessible to a wider global readership. This is a very useful addition to the global literature on research into eye health provision.

Is the work clearly and accurately presented and does it cite the current literature?

Partly

If applicable, is the statistical analysis and its interpretation appropriate?

Yes

Are all the source data underlying the results available to ensure full reproducibility?

No

Is the study design appropriate and is the work technically sound?

Partly

Are the conclusions drawn adequately supported by the results?

Partly

Are sufficient details of methods and analysis provided to allow replication by others?

Partly

Reviewer Expertise:

Optometry, Eye health, Survey Design and analysis, Psychology,

I confirm that I have read this submission and believe that I have an appropriate level of expertise to confirm that it is of an acceptable scientific standard.

# Reviewer report: Reviewer response for version 1

**DOI:** 10.5256/f1000research.174818.r397069

**Published:** 2025-08-22

**Authors:** Benoit Tousignant

**Affiliations:** 1 University of Montreal, Montreal, Canada

The authors present an interesting study on the knowledge and practice levels of primary care nurses with regard to eye care in rural South Africa.  Some revisions are required to strengthen the manuscript.

 - "African race" is used throughout abstract and manuscript.  consider reviewing and referring to specific South African ethnicities of the participants, rather than a too general term

 - references should include WHO World report on vision (2019), in addition to refs 1-5

 - instead of ref 6, use WHO Eye care competency framework.  Adapt and rephrase manuscript accordingly, using wording form that document.

 - In Study design, when first mentioning the PI, include that person's initials in parentheses

 - Data collection: 2nd line, missing word: "...at most sites due TO absenteeism"

 - a copy of the questionnaire is required

 - methods should specify how analysis was made with Likert: how were neutral responses handled?

 - Results section generally: the text should briefly report highlights of results and then refer to Tables 3-4-5 for the complete results.

 - Tables 3 and 5: add note to say which stat test the p-values refer to (chi-square?)

 - methods (relating to results presented in Table 5: include example of cataract (or other condition) pictures used to test knowledge and practice?  how were these pictures or knowledge items selected, and by whom?

 - manuscript would benefit from a deeper analysis, including additional analyses such as looking at associations between % of correct knowledge and age, sex, type of institution, job title, duration of service. Use non-parametric tests as need if cell counts are below 5.  Repeat these analyses for % of correct practice.  Report carefully, given the small numbers of certain cells/subgroups.

Is the work clearly and accurately presented and does it cite the current literature?

Partly

If applicable, is the statistical analysis and its interpretation appropriate?

Partly

Are all the source data underlying the results available to ensure full reproducibility?

No

Is the study design appropriate and is the work technically sound?

Partly

Are the conclusions drawn adequately supported by the results?

Partly

Are sufficient details of methods and analysis provided to allow replication by others?

Partly

Reviewer Expertise:

Eye care, public health, Global health

I confirm that I have read this submission and believe that I have an appropriate level of expertise to confirm that it is of an acceptable scientific standard, however I have significant reservations, as outlined above.

# Response

**Published:** 2026-01-17

**Authors:** Zamadonda Xulu-Kasaba

**Affiliations:** Optometry, University of KwaZulu-Natal - Westville Campus, Durban, KwaZulu-Natal, South Africa

***No.***

***Query***

 Response to Query

***1***

***The authors present an interesting study on the knowledge and practice levels of primary care nurses with regard to eye care in rural South Africa.  Some revisions are required to strengthen the manuscript.***

***- In Study design, when first mentioning the PI, include that person's initials in parentheses***

 Initials of the PI have been included with the first mention.

***2***

***- "African race" is used throughout abstract and manuscript.  consider reviewing and referring to specific South African ethnicities of the participants, rather than a too general term***

***- references should include WHO World report on vision (2019), in addition to refs 1-5***

***- instead of ref 6, use WHO Eye care competency framework.  Adapt and rephrase manuscript accordingly, using wording form that document.***

 African is the least offensive term that is agreeable with ethnic people in Africa generally. This is why it was preferred in relation to “Blacks”. Other terms would have been tribalistic, which is a contentious issue now in South Africa. As such, “African” was the best option.

 Ref 2 (2021) contains updated data from the World report on vision (2019). We opted to use the latest available values.

***3***

***- Data collection: 2nd line, missing word: "...at most sites due TO absenteeism"***

 Thank you, corrected.

***4***

***- a copy of the questionnaire is required***

 At the end of the manuscript, under “Data availability statement” there is a link that leads to a repository with the questionnaire and other data for the study. The journal asks that it be placed in this online repository.

***- methods should specify how analysis was made with Likert: how were neutral responses handled?***

 Thank you, this has been addressed with the addition of a paragraph detailing it at the end of the methods section.

***- Results section generally: the text should briefly report highlights of results and then refer to Tables 3-4-5 for the complete results.***

 Noted, thank you.

***- Tables 3 and 5: add note to say which stat test the p-values refer to (chi-square?)***

 Noted and addressed.

***- methods (relating to results presented in Table 5: include example of cataract (or other condition) pictures used to test knowledge and practice?  how were these pictures or knowledge items selected, and by whom?***

 As detailed under “Data collection, processing and analysis”, the tool was developed using various primary health guidelines and screening documents. Coloured pictures were selected from those documents by the researchers in the study. These are all in the suppository and available for anyone to see. Next to each picture was a statement on naming the condition e.g. this is a mature cataract accompanied by the 5 category Likert Scale, then there would be a second question e.g. It requires an immediate transfer to a hospital, with the respondent indicating again from Strongly agree to disagree.

***- manuscript would benefit from a deeper analysis, including additional analyses such as looking at associations between % of correct knowledge and age, sex, type of institution, job title, duration of service. Use non-parametric tests as need if cell counts are below 5.  Repeat these analyses for % of correct practice.  Report carefully, given the small numbers of certain cells/subgroups.***

 Tables 3, 4 and 5 have given most of the suggested information. Associations relating to gender, type of institution and job title became redundant when we tried them due to the fact that almost 100% of the participants shared the same characteristics e.g. all were females, working at the same type of institution with the same job title. Otherwise, thank you for the input, it is worth remembering for future data analysis and discussion.

# Reviewer report: Reviewer response for version 1

**DOI:** 10.5256/f1000research.174818.r402133

**Published:** 2025-08-18

**Authors:** Sakshi Chaturvedi

**Affiliations:** 1 Banasthali Vidyapith, Rajasthan, India

The study falls short in addressing nurses' knowledge of eye health, a critical area that directly influences the quality and timeliness of patient care. Without establishing this foundational context, the introduction misses an opportunity to highlight the broader implications of inadequate ocular awareness among nursing professionals. Furthermore, the tools used to assess knowledge and practices appear to lack sufficient validation, raising concerns about the reliability and interpretability of the results. The sampling technique is also vaguely described, with limited information on how participants were selected or whether the sample is representative factors that are essential for ensuring methodological rigor and generalizability.

Is the work clearly and accurately presented and does it cite the current literature?

Partly

If applicable, is the statistical analysis and its interpretation appropriate?

Partly

Are all the source data underlying the results available to ensure full reproducibility?

Yes

Is the study design appropriate and is the work technically sound?

Yes

Are the conclusions drawn adequately supported by the results?

Yes

Are sufficient details of methods and analysis provided to allow replication by others?

Yes

Reviewer Expertise:

Nursing research , Public health , Nursing education

I confirm that I have read this submission and believe that I have an appropriate level of expertise to confirm that it is of an acceptable scientific standard, however I have significant reservations, as outlined above.

## References

[1] : Need to Educate Student Nurses for Eye Care: A Descriptive Analysis of Student Nurses’ eye health literacy. *International Journal of Nursing Education* .2023;15(4) : 10.37506/ijone.v15i4.20043 52-58 10.37506/ijone.v15i4.20043

# Response

**Published:** 2026-01-17

**Authors:** Zamadonda Xulu-Kasaba

**Affiliations:** Optometry, University of KwaZulu-Natal - Westville Campus, Durban, KwaZulu-Natal, South Africa

***1***

***The study falls short in addressing nurses' knowledge of eye health, a critical area that directly influences the quality and timeliness of patient care. Without establishing this foundational context, the introduction misses an opportunity to highlight the broader implications of inadequate ocular awareness among nursing professionals.***

 This is what the study sought to assess, the knowledge of nurses on eye health and their practices. As such, it was not possible to discuss what the study was trying to assess in the introduction. This is why the knowledge is discussed under the Discussion section, after results had been obtained.

***2***

***Furthermore, the tools used to assess knowledge and practices appear to lack sufficient validation, raising concerns about the reliability and interpretability of the results.***

 Albeit not statistically validated, all tools were piloted in another province as stated in the Methodology section under “Data collection, processing and analysis”. Through this process, amendments were made, the tool refined and finalised for the study.

# Reviewer report: Reviewer response for version 1

**DOI:** 10.5256/f1000research.174818.r361697

**Published:** 2025-02-04

**Authors:** Michael Bowen

**Affiliations:** 1 The College of Optometrists, London, England, UK

**Study Setting section:** It would be useful to include details of the number of Primary Health Care clinics in KwaZulu-Natal, which is used as the comparison province to the study province.

 The use of "AND" as the acronym for Alfred Nzo District Municipality is potentially odd for readers less familiar with this acronym. For may English speakers AND looks like a capitalized "and". It might be helpful to change to using "ANDM" as the acronym.

**Study Design section:** in this section there is a statement that "a figure of 50% was used for the knowledge base" - this is somewhat unclear. Does this mean that respondents were assumed (for the purposes of the power calculations) to be likely to answer 50% of the questions correctly? This would benefit from some clarification.

**Results section:** *Table 3* is titled "Basic knowledge on roles of different Human resources for eye health (HReH)" however, it is not clear that the data being reported relate to an objective assessment of knowledge. Several of the questions for which response data are reported here are reporting participants view of their own knowledge. It would be preferable to re-title and ensure that there is some text added to clarify the nature of these data. They are still of interest - but need to be accurately described.

 The methods section mentions a 5 point Likert scale - it is not clear from any of the tables which data were derived from these questions. Ideally a copy of the questionnaire used should be included as an annex or within supplementary materials. It is slightly difficult to review the paper without seeing the questionnaire.

 Table 5 - this table reports data relating to participants ability to correctly identify eye health conditions from photographs. It was not clear from the methods section whether these photographs had been tested by a group of eye health professionals (such as optometrists / ophthalmologists) to provide a base-line for the rate at which qualified eye health professionals were able to correctly identify the conditions from the images. This is important as it provides some validation that the images are 'good enough' representations of the conditions in question. It would be helpful to share some of the images used in the paper or as supplementary material.

 Table 5 - It is not clear from the table or the text in the results section whether the data relating to the %ages of participants who made the correct management responses relate to the whole group or just to those who were able to correctly identify the condition. This is important to the interpretation of these data, so would be helpful to make this clear. For example, for Uveitis, if 16% of the participants correctly identified it as Uveitis, but only 32% of those who correctly identified it knew that it needed an urgent referral, that provides different insights than if if 16% correctly identified the Uveitis, but 32% of all the participants knew it needed an urgent referral. In the latter case, even though they didn't know what the condition was an additional 16% of participants knew that it needed urgent referral.

 Table 5 - Primary Open Angle Glaucoma - it is unclear from the table, or from the information available elsewhere in the paper, what information was actually given to participants to test their ability to identify POAG and how the information given would lead to the correct management being no referral - monitor yearly. The information summarised in the table suggests that 'throbbing eye pain' was one of the symptoms offered to participants, which does not necessarily suggest stable POAG or OHT. It is also unclear how any of the PHC's in the study would be able to safely monitor OHT or stable POAG, given their lack of equipment. This question and the associated data need to be reviewed by the authors and their inclusion in the final paper considered carefully.

 Discussion section:

 Page 7, paragraph 3: "...one of the higher prevalence's" (a) if prevalences is to be used, the apostrophe needs to be removed; (b) I am not sure that it is helpful to use "prevalences" in reference to the number of qualified nurses in the work force in a region. I would suggest editing this to read: "....one of the higher proportions of qualified to unqualified nurses in the country."

 Same paragraph: With reference to the participants mainly speaking isiXhosa, it would be useful to readers to include some information about the population in that region - was the percentage of isiXhosa speakers among the participant sample representative of the rates of isiXhosa speakers in the population of ANDM?

 Page 8, paragraph 2: the second sentence in this paragraph is confusing: "The number of participants was lessened by the fact that the original sample size was incorrect, owing to poor record keeping". This is not really correct. The original population size was incorrect due to the records of nursing staff being inaccurate. While this error could have led to an incorrect sample size being calculated, the investigators took appropriate measures to establish a more reliable / accurate estimate of the population size, and thus were able to derive a more reliable sample size. However, since it appears that the authors intention in this section is to address the concerning lack of accurate records for nursing staff numbers, I would suggest simply rewording this along the lines of: "The challenges encountered by the research team in this project in seeking to establish a reliable estimate of the total number of nursing staff employed within PHCs across ANDM are noteworthy. The initial population number provided was nearly double the final population established by the researchers."

 In the same paragraph the sentence: "Consequently, this insufficient staffing worsens the burden of diseases, increases VI and the prevalence of avoidable blindness..." while likely to be true, for a research paper it is slightly hyperbolic. It would be preferable to reword along the lines of: "The very low levels of PHC staff seems likely to contribute to increased levels of VI and preventable sight loss / blindness, and potentially a worse burden of disease across the province, as it is hard to see how such low levels of PHC staffing would fail to lead to reduced service delivery / access to care. These low staffing levels are likely to make it much harder to achieve universal health coverage in a meaningful way."

 Page 8 - Eye health knowledge section: First paragraph, final sentence "...that preventable blindness is addressed timeously." "Timeously" should be replace by "in a timely way".

 Second paragraph of Page 8 - "Table 3 shows..." in this this paragraph the authors suggest that the poor awareness of the various eye health professions' roles "...indirectly affects referral practices...". It is not clear how the data from this research supports this assertion. If this is not a direct finding of the research. For example - was there an analysis of the data that showed that those who incorrectly identified the competencies and roles of the different eye health professionals performed less well on the questions relating to making correct management decisions where these related to referral? In any case, lower knowledge in this area seems highly likely to directly affect referral practices - this could be offered as an observation if there is not a specific analysis to support this as a "finding" per se.

 Third paragraph in this section, starting with "Knowledge of common eye conditions..." in this first sentence "were" needs to be replaced with "was".

 Page 8, final paragraph: I am not sure it is helpful to refer to the "prevalence" of a poor capacity of participants to correctly identify cataract - this should be reworded along the lines of: "The low level of knowledge of cataract in this study is significantly lower than..."

 Same paragraph - the grammar in the sentence on Foreign body sensations is a little odd - suggest rewording along the lines of: "'Foreign body sensations' was the only condition which, although not correctly identified by the majority of participants, produced appropriate management responses from  an acceptable proportion of participants".

 Training in eye care section: it is unfortunate that the analyses of the data reported here do no include any analysis of whether the nurses who HAD received some eye health training did in fact perform better on the identification and management tasks, as well as on the awareness and perception of other eye health professions competencies. While the numbers who received training may have been too small to support a fully reliable sub-group analysis, this seems too important a question not to at least investigate to some extent, even if in reporting the low numbers in the "with training" group had to be raised as a cautionary factor.

 The reference to "There were poor management guidelines in place and no set referral criteria used" appears to introduce / refer to data or information not reported earlier in the paper. It would certainly be interesting to have seen data relating to the management guidelines used in the PHCs that participated in the study, as well as they referral criteria for those sites. Without data, this assertion lacks the impact it could have otherwise.

 Second paragraph in this section: the sentence starting: "It stood to reason that the Kenyan study...." this sentence is somewhat muddled in the section "...which could be cited as an example of competence overreaching as those that were not trained learned from the others and managed patients satisfactorily." I think that the authors mean that the Kenyan study could be cited as an example of where the training of some staff lead to improved performance by all the staff due to the untrained staff learning from those who had received training - if this is the case, that sentence would benefit from rewording.

 Clinical skill and observation and practice norms: the first sentence in this section seems potentially problematic. Since this research did not compare 'community / primary care nurses' generally in South Africa or elsewhere with the PHC nursing staff, it seems perhaps unreasonable to expect them to know how to do even a basic refraction examination, especially since almost none of them have had any eye health training either in their training to qualification or post-qualification. How are the research team defining "the expected level"? When and where was this level established / agreed and how was it communicated to nursing or leadership staff in the PHC system within ANDM? I think it would be preferable to reword the first two sentences for this section along the lines of: 

 "The study highlighted that current nursing staff within the ANDM PHC system lack the ability to carry out a basic visual acuity assessment, which should be a core requirement for the PHC clinics to be able to provide. Our data showed that the majority of participants in this study scored 6/10 or less on this clinical technique for assessing visual acuity."

 Within the recommendations section it would be helpful to consider adding something about establishing clear referral guidelines and pathways (which the authors mention earlier in the text) and developing and providing all PHC clinics with eye health diagnostic guides and management guidance. It would seem feasible for an "eye health pack" to be developed, to include Snellen (or even LogMAR) and tumbling E charts, tape measures, occluders, vision assessment check lists (perhaps as posters for display for ease of staff use) and so on.

Is the work clearly and accurately presented and does it cite the current literature?

Partly

If applicable, is the statistical analysis and its interpretation appropriate?

Yes

Are all the source data underlying the results available to ensure full reproducibility?

No

Is the study design appropriate and is the work technically sound?

Partly

Are the conclusions drawn adequately supported by the results?

Partly

Are sufficient details of methods and analysis provided to allow replication by others?

Partly

Reviewer Expertise:

Optometry, Eye health, Survey Design and analysis, Psychology,

I confirm that I have read this submission and believe that I have an appropriate level of expertise to confirm that it is of an acceptable scientific standard, however I have significant reservations, as outlined above.

# Response

**Published:** 2026-01-17

**Authors:** Zamadonda Xulu-Kasaba

**Affiliations:** Optometry, University of KwaZulu-Natal - Westville Campus, Durban, KwaZulu-Natal, South Africa

***1.*** ***It would be useful to include details of the number of Primary Health Care clinics in KwaZulu-Natal, which is used as the comparison province to the study province.***

 KwaZulu-Natal is a province whilst Alfred Nzo District is part of a province. As such, it would be incorrect to relate the two. This is why a district that is similar in its socio-economic status was used as a comparison to the district of the study.

***2.*** ***The use of "AND" as the acronym for Alfred Nzo District Municipality is potentially odd for readers less familiar with this acronym. For may English speakers AND looks like a capitalized "and". It might be helpful to change to using "ANDM" as the acronym.***

 Makes sense, this has been considered *.*

***Study Design section: in this section there is a statement that "a figure of 50% was used for the knowledge base" - this is somewhat unclear. Does this mean that respondents were assumed (for the purposes of the power calculations) to be likely to answer 50% of the questions correctly? This would benefit from some clarification.***

 This was an error. It has been corrected under data analysis.

**Results section:** ***Table 3* is titled "Basic knowledge on roles of different Human resources for eye health (HReH)" however, it is not clear that the data being reported relate to an objective assessment of knowledge. Several of the questions for which response data are reported here are reporting participants view of their own knowledge. It would be preferable to re-title and ensure that there is some text added to clarify the nature of these data. They are still of interest - but need to be accurately described.**

 The rationale behind this question simply relates accurate knowledge resulting in relevant referral and triage, which has been explained in the discussion. Knowing the main role of an Ophthalmologist (surgery) and an Optometrist (vision correction), for example, means that one would know that a patient with a cataract needs to be referred to an ophthalmologist as the only correction in that instance is surgery. One would have to establish the participants’ own knowledge.

***The methods section mentions a 5 point Likert scale - it is not clear from any of the tables which data were derived from these questions. Ideally a copy of the questionnaire used should be included as an annex or within supplementary materials. It is slightly difficult to review the paper without seeing the questionnaire.***

 A new paragraph has been added in the methodology to improve on the Likert scale. Furthermore, please see the bottom of the manuscript where data is being declared. The journal requested that this be placed in a zenodo repository space. The zenodo link is provided in that section, and the questionnaires are all availed there for perusal and review.

***Table 5 - this table reports data relating to participants ability to correctly identify eye health conditions from photographs. It was not clear from the methods section whether these photographs had been tested by a group of eye health professionals (such as optometrists / ophthalmologists) to provide a base-line for the rate at which qualified eye health professionals were able to correctly identify the conditions from the images. This is important as it provides some validation that the images are 'good enough' representations of the conditions in question. It would be helpful to share some of the images used in the paper or as supplementary material.***

 In the methods section we included information stating that the images were taken from documents already used within the Departments and in their training manuals. Further to this, the images were supplied in high resolution colour (as you will see in the questionnaire). As such, we believe that the quality was sufficient for the study. All of this has been supplied as supplementary material in the repository.

***Table 5 - It is not clear from the table or the text in the results section whether the data relating to the %ages of participants who made the correct management responses relate to the whole group or just to those who were able to correctly identify the condition. This is important to the interpretation of these data, so would be helpful to make this clear. For example, for Uveitis, if 16% of the participants correctly identified it as Uveitis, but only 32% of those who correctly identified it knew that it needed an urgent referral, that provides different insights than if if 16% correctly identified the Uveitis, but 32% of all the participants knew it needed an urgent referral. In the latter case, even though they didn't know what the condition was an additional 16% of participants knew that it needed urgent referral.***

 All the results report on the entire group. Example, if 16% reported uveitis correctly and 32% thought it needed urgent referral it means that there is a 16% participant population that would still refer for urgent attention even though they do not know that this is uveitis. As this picture was accompanied by the definition of pain, the respondents might have concluded that even though the condition was not identified correctly, the symptom of pain warranted an urgent referral.

***Table 5 - Primary Open Angle Glaucoma - it is unclear from the table, or from the information available elsewhere in the paper, what information was actually given to participants to test their ability to identify POAG and how the information given would lead to the correct management being no referral - monitor yearly. The information summarised in the table suggests that 'throbbing eye pain' was one of the symptoms offered to participants, which does not necessarily suggest stable POAG or OHT. It is also unclear how any of the PHC's in the study would be able to safely monitor OHT or stable POAG, given their lack of equipment. This question and the associated data need to be reviewed by the authors and their inclusion in the final paper considered carefully.***

 Noted with thanks. Please take a look at the data collection tool for clarity with the actual data given.

***Discussion section:***

***Page 7, paragraph 3: "...one of the higher prevalence's" (a) if prevalences is to be used, the apostrophe needs to be removed; (b) I am not sure that it is helpful to use "prevalences" in reference to the number of qualified nurses in the work force in a region. I would suggest editing this to read: "....one of the higher proportions of qualified to unqualified nurses in the country."***

***Same paragraph: With reference to the participants mainly speaking isiXhosa, it would be useful to readers to include some information about the population in that region - was the percentage of isiXhosa speakers among the participant sample representative of the rates of isiXhosa speakers in the population of ANDM?***

 Thank you, these errors have been corrected. The revised version suggests that some of the nurses are not qualified, which would be an incorrect statement.

 In the sentence that states that most participants spoke IsiXhosa, we later expressed that in that province IsiXhosa is the main language. As such, this would support representivity of the data.

***Page 8, paragraph 2: the second sentence in this paragraph is confusing: "The number of participants was lessened by the fact that the original sample size was incorrect, owing to poor record keeping". This is not really correct. The original population size was incorrect due to the records of nursing staff being inaccurate. While this error could have led to an incorrect sample size being calculated, the investigators took appropriate measures to establish a more reliable / accurate estimate of the population size, and thus were able to derive a more reliable sample size. However, since it appears that the authors intention in this section is to address the concerning lack of accurate records for nursing staff numbers, I would suggest simply rewording this along the lines of: "The challenges encountered by the research team in this project in*** ***seeking to establish a reliable estimate of the total number of nursing staff employed within PHCs across ANDM are noteworthy. The initial population number provided was nearly double the final population established by the researchers."***

 Thank you, this has been amended.

***In the same paragraph the sentence: "Consequently, this insufficient staffing worsens the burden of diseases, increases VI and the prevalence of avoidable blindness..." while likely to be true, for a research paper it is slightly hyperbolic. It would be preferable to reword along the lines of: "The very low levels of PHC staff seems likely to contribute to increased levels of VI and preventable sight loss / blindness, and potentially a worse burden of disease across the province, as it is hard to see how such low levels of PHC staffing would fail to lead to reduced service delivery / access to care. These low staffing levels are likely to make it much harder to achieve universal health coverage in a meaningful way."***

 Thank you this was amended.

***Page 8 - Eye health knowledge section: First paragraph, final sentence "...that preventable blindness is addressed timeously." "Timeously" should be replace by "in a timely way".***

***Second paragraph of Page 8 - "Table 3 shows..." in this this paragraph the authors suggest that the poor awareness of the various eye health professions' roles "...indirectly affects referral practices...". It is not clear how the data from this research supports this assertion. If this is not a direct finding of the research. For example - was there an analysis of the data that showed that those who incorrectly identified the competencies and roles of the different eye health professionals performed less well on the questions relating to making correct management decisions where these related to referral? In any case, lower knowledge in this area seems highly likely to directly affect referral practices - this could be offered as an observation if there is not a specific analysis to support this as a "finding" per se.***

 This was addressed above when explaining why nurses needed to know the main roles of different HReH.

***Third paragraph in this section, starting with "Knowledge of common eye conditions..." in this first sentence "were" needs to be replaced with "was".***

 Amended, thank you.

***Page 8, final paragraph: I am not sure it is helpful to refer to the "prevalence" of a poor capacity of participants to correctly identify cataract - this should be reworded along the lines of: "The low level of knowledge of cataract in this study is significantly lower than..."***

***Same paragraph - the grammar in the sentence on Foreign body sensations is a little odd - suggest rewording along the lines of: "'Foreign body sensations' was the only condition which, although not correctly identified by the majority of participants, produced appropriate management responses from  an acceptable proportion of participants".***

 Duly noted and amended where applicable.

***Training in eye care section: it is unfortunate that the analyses of the data reported here do no include any analysis of whether the nurses who HAD received some eye health training did in fact perform better on the identification and management tasks, as well as on the awareness and perception of other eye health professions competencies. While the numbers who received training may have been too small to support a fully reliable sub-group analysis, this seems too important a question not to at least investigate to some extent, even if in reporting the low numbers in the "with training" group had to be raised as a cautionary factor.***

***The reference to "There were poor management guidelines in place and no set referral criteria used" appears to introduce / refer to data or information not reported earlier in the paper. It would certainly be interesting to have seen data relating to the management guidelines used in the PHCs that participated in the study, as well as they referral criteria for those sites. Without data, this assertion lacks the impact it could have otherwise.***

***Second paragraph in this section: the sentence starting: "It stood to*** ***reason that the Kenyan study...." this sentence is somewhat muddled in the section "...which could be cited as an example of competence overreaching as those that were not trained learned from the others and managed patients satisfactorily." I think that the authors mean that the Kenyan study could be cited as an example of where the training of some staff lead to improved performance by all the staff due to the untrained staff learning from those who had received training - if this is the case, that sentence would benefit from rewording.***

***Clinical skill and observation and practice norms: the first sentence in this section seems potentially problematic. Since this research did not compare 'community / primary care nurses' generally in South Africa or elsewhere with the PHC nursing staff, it seems perhaps unreasonable to expect them to know how to do even a basic refraction examination, especially since almost none of them have had any eye health training either in their training to qualification or post-qualification. How are the research team defining "the expected level"? When and where was this level established / agreed and how was it communicated to nursing or leadership staff in the PHC system within ANDM? I think it would be preferable to reword the first two sentences for this section along the lines of:***

***"The study highlighted that current nursing staff within the ANDM PHC system lack the ability to carry out a basic visual acuity assessment, which should be a core requirement for the PHC clinics to be able to provide. Our data showed that the majority of participants in this study scored 6/10 or less on this clinical technique for assessing visual acuity."***

 All noted and addressed where necessary in the revised manuscript. Other input is valuable and is most appreciated.

***Within the recommendations section it would be helpful to consider adding something about establishing clear referral guidelines and pathways (which the authors mention earlier in the text) and developing and providing all PHC clinics with eye health diagnostic guides and management guidance. It would seem feasible for an "eye health pack" to be developed, to include Snellen (or even LogMAR) and tumbling E charts, tape measures, occluders, vision assessment check lists (perhaps as posters for display for ease of staff use) and so on.***

 Added suggestions as per recommendation.
